# Clinico-behavioral, hepato-renal, erythrocytic, and genotoxicity induced by sub-chronic oral doses of acetochlor in male Japanese quail

**DOI:** 10.1016/j.psj.2026.107059

**Published:** 2026-05-01

**Authors:** Dong Shiqi, Mohammed Al-Rasheed, Sahar Faiz Lodhi, Nuzhat Sial, Riaz Hussain, Maha Abdullah Momenah, Mahmoud H.A. Mohamed, Ahmed M.A. Meligy, Mohamed S. Ahmed, Ahmed Alfifi, Nahid Abdelraheem Ali, Zhang Jiabin

**Affiliations:** a College of Animal Science and Technology, Tarim University, Aral, 843300, China; b Department of Clinical Sciences, College of Veterinary Medicine, King Faisal University, Al-Ahsa, P.O. Box 400, 31982, Saudi Arabia; c Department of Zoology, The Islamia University of Bahawalpur, 63100, Pakistan; d Department of Pathology, Faculty of Veterinary and Animal Sciences, The Islamia University of Bahawalpur, 63100, Pakistan; e Department of Biology, College of Science, Princess Nourah bint Abdulrahman University, P.O. Box 84428, Riyadh 11671, Saudi Arabia; f Department of Clinical Science, Central Lab, College of Veterinary Medicine, King Faisal University, P.O. Box: 400, Al-Ahsa, 31982, Saudi Arabia; g Department of Public Health, College of Veterinary Medicine, King Faisal University, Al-Ahsa, P.O. Box 400, 31982, Saudi Arabia; h Department of Biology, College of Science, King Khalid University, Abha, 62521, Saudi Arabia

**Keywords:** Acetochlor, Japanese quail, Genotoxicity, Oxidative stress, Histopathology

## Abstract

Acetochlor is one of the well-known and frequently used herbicides that acts as a potent endocrine disruptor. Still, there is a scarcity of studies examining the adverse effects of acetochlor on birds. Therefore, this study evaluated the behavioral alterations, oxidative stress, antioxidant enzymes, genotoxicity and histopathological impacts of subchronic oral doses of acetochlor in quails. A total of 80 male quails were procured and reared in four groups (A-D) for 45 days. Group A served as the control, while the birds reared in groups B, C and D received 20 mg/kg, 30 mg/kg and 40 mg/kg acetochlor on daily basis, respectively, for 45 days. Different clinical and behavioral signs were observed in quails exposed to higher doses of acetochlor. The quantity of antioxidant enzymes, including superoxide dismutase (SOD), reduced glutathione (GSH), and catalase (CAT), was significantly reduced (P < 0.05) in treated birds compared to normal/untreated birds. The values of oxidative stress parameters like lipid peroxidase (LPO) increased significantly (P ≤ 0.05) in the liver of Japanese quails exposed to higher concentrations of acetochlor. Significantly (P ≤ 0.05) increased values of DNA damage in isolated hepatocytes, enterocytes, and cells of the kidneys were recorded in acetochlor-treated quails. Results on the formation of micronuclei in erythrocytes revealed significantly (P ≤ 0.05) higher percentile rate in birds treated with higher concentrations of herbicide compared to untreated birds. At the histopathological level, various microscopic lesions like necrosis, disorganization, and disruption of hepatic cords in the liver, degeneration of renal tubular cells, widening of urinary space, necrosis of renal tubules in the kidneys, and degeneration and depletion of splenic cells in the spleen of quails were examined. The results of this research highlighted that Acetochlor induces different pathological ailments in Japanese quail even at sub-chronic oral doses.

## Introduction

Pesticides are synthetic compounds that are extensively used in agro-production units to enhance the yield of food crops and to control different disease-causing vectors in public health (Yang et al., 2021; Al-Saeed et al., 2023). After applications, the pesticides contaminate soil, water, and food crops. The frequent and extensive use of herbicides has raised huge environmental concerns, causing adverse effects on the wildlife, aquatic ecosystem and public health (Ghaffar et al., 2015; Naseem et al., 2022). Persistent and occupational contact with different industrial and agrochemicals, including insecticides, herbicides and pesticides, causes numerous toxic effects on different ecosystems leading to increased health problems such as neurodegeneration, premature delivery, cancer, infertility, abortion, and various congenital ailments (Zahoor et al., 2022; Altaf et al., 2025; Boonkhao et al., 2025). It has been recorded that monitoring and evaluation of various synthetic chemicals especially pesticides and insecticides is of useful and vital importance because these chemicals are considered as highly toxic to a variety of terrestrial and aquatic animals and induce several health problems like neurodegenerative disorders, cancer and various chronic health effects (Muhyuddin et al., 2025). Avian species having a distinctive place in terrestrial ecosystem (Fatima et al., 2025, Iqbal et al., 2025) are considered the best indicators of a healthy environment and can reflect a reliable and early warning for the monitoring of environmental and health hazards pollutants (Hussain et al., 2019; Ouyang et al., 2021; Rani et al., 2023; Salam et al., 2025). Intensification of the agricultural sector has involved the extensive use of pesticides (DiBartolomeis et al., 2019) over the last few decades and is persistently damaging both terrestrial and aquatic life, including birds. The extensive and indiscriminate application of various agrochemicals has tremendously increased in public health management, agriculture, veterinary practices, and other industries during the past few decades (Naseem et al., 2022; Shamgunov et al., 2025). The deleterious and toxic effects due to extensive use of a variety of insecticides, pesticides, fungicides and herbicides in agricultural and allied sectors have become a matter of great concern due to their quick dispersal in water bodies, air, food crops and various other food products obtained from animals, vegetables and cereal crops (Raza et al., 2022). The animals living in terrestrial and aquatic environments are at great risk due to persistent contaminations (Mahmood et al., 2022). Herbicide contamination has been a major concern in various countries due to inadequate regulations and/or their enforcement (Dessouki et al., 2012), leading to poisoning in both humans and wildlife through polluted food and water. Herbicides are extensively used in both urban and agricultural zones, leading to their increased presence along with negative effects on non-target organisms (Ulrich et al., 2018).

Acetochlor is used as a selective herbicide primarily for the control of annual grasses and broad-leaf weeds in different food crops including corn, soybean, peanut, wheat and cotton. It kills the broad-leaf weeds by inhibiting the process of growth via disruption of capability of essential proteins production (Zhang et al., 2023). It has been recorded that acetochlor causes negative effects on non-target animals, including invertebrates, fish, amphibians, birds, and mammals. According to a previous study, exposure to more than 20 mg/kg of acetochlor (ACT) caused a significant decrease in the growth and reproduction of earthworms as well as induced DNA damage in these organisms (Xiao et al., 2006). Different studies have shown that ACT can induce oxidative stress and decrease the quantity of antioxidative enzymes in the liver of zebrafish (*Danio rerio*) and induce liver carcinoma in humans (Chang et al., 2020; Huang et al., 2020). It is critical to investigate the transfer and accumulation of different environmental pollutants in the visceral tissues of animals to establish an appropriate toxicity threshold level (Wu et al., 2021). Acetochlor is prevalent in water and soil ecosystems and has been frequently detected (11,400 ng/L) in natural environments across the globe (Rebich et al., 2004). Studies have also reported high concentrations (10–100 ng/L) of acetochlor in aquatic ecosystem in United States (Scribner et al., 2000; Hladik et al., 2008). Moreover, 0.03–709.37 μg/kg of acetochlor in soil from northeastern China, 54.76 μg/kg residual concentrations in maize land, 61.36 μg/kg riparian soils and 2.53 μg/kg in sediments (Sun et al., 2011) has been detected. Acetochlor is persistently utilized in different industries, which not only increases mortality, influences feeding behavior, but also induces endocrine disorders (Xue et al., 2020), anomalous gene expression (Li et al., 2018), and oxidative stress in exposed animals, leading to abnormal development, abnormal structures of DNA, and damage to different visceral organs (Xue et al., 2020). It has been recorded that monitoring and investigation of biochemical parameters, including oxidative stress, antioxidant enzymes, and mutagenic potential, are useful tools to assess the mechanisms of toxicity of different pesticides and herbicides (Al-Saeed et al., 2023). Therefore, the current experimental research was conducted to investigate the mechanisms of toxicity of acetochlor in male Japanese quails.

## Materials and methods

### Experimental design

A total of 80 male Japanese quails were obtained from the commercial market and were placed in wooden cages. After acclimatization, the quail were then divided into four groups (A, B, C, and D), and twenty (n = 20) quail were kept in each group. The birds were fed commercial poultry feed for a period of 45 days. Acetochlor was orally administered to quails of groups (B, C, and D) daily. The birds placed in group A served as untreated/normal control and did not receive acetochlor. The quails of groups B, C, and D received 20mg/kg, 30mg/kg, and 40mg/kg of acetochlor mixed in water, respectively. The birds were monitored daily for any visible changes in behavior or clinical symptoms. For tissue collection, five (n = 5) quails were randomly picked from each group and were killed on days 15, 30, and 45 of the experiment. No mortalities were reported during the experiment.

### Ethics Statement

All the experiments were conducted according to the guidelines of the Animal Ethics Committee of the Islamia University of Bahawalpur (approval No. AEC-24-13).

### Oxidative stress parameters and status of antioxidant enzymes

To investigate the biochemical parameters, birds from the control and treatment groups were killed and dissected on days 15, 30, and 45 of the experiment. The birds were slaughtered for the collection of tissues without using anesthesia according to the previous study (Arshad et al., 2024). Briefly, the bird’s legs are with one hand supporting the body. Then we grasped the head behind the skull with fingers, while pulling the head down to stretch the neck and separate the skull from the atlas-vertebra. The skin of animals was incised from the sternum to the pubis to expose the peritoneal cavity. Liver tissues from five birds reared in control and acetochlor-treated groups were separately obtained at days 15, 30, and 45 of the experiment at the time of necropsy for biochemical analyses. All the collected liver tissues were immediately placed in chilled normal saline solution, and then a homogenate was prepared separately from each liver tissue for the measurement of different antioxidant enzymes and oxidative stress profile. The enzymatic antioxidants GSH (mmol/g tissue), CAT (units/min), and SOD (units/mg protein) were measured in liver tissues. Following the earlier published investigations, oxidative stress indices, including LPO by measuring thiobarbituric acid reactive substances (TBARS; nmol/TBARS formed/ mg protein/min) in liver tissues, were recorded (Ghannam et al., 2025). Different enzymatic antioxidants such as SOD (Rashid et al., 2025), GSH, and CAT (Gill et al., 2025) were quantified in the liver tissues of the treated and control birds using a UV-spectrophotometer.

### Evaluation of genotoxicity

The alkaline assay, also known as single cell gel electrophoresis (comet assay) protocol as described by (Singh et al., 1988) was used to estimate the DNA damage potential of herbicide in isolated cells of different tissues (Liver, kidneys, and intestine). Briefly, the separated visceral organs were minced and suspended in Hank’s Balanced salt solution with 0.75 M NaCl and 0.24 M Na_2_EDTA at a concentration of 1g/ml for isolation of cells. After that, the samples were centrifuged, and the cells were removed in the form of pellets. To prepare the slides, 0.5% agarose was layered on frosted slides, followed by a second layer of low-melting agarose containing cells. The slides were kept in the refrigerator for the solidification of layers. Finally, a third layer of agarose (0.8%) was added on top of the second layer, followed by a cover slip. Then the slides were placed in chilled lysing solution in a refrigerator in a dark condition for one hour and then transferred to a horizontal electrophoresis tank which was filled with electrophoresis buffer. Then, electrophoresis was carried out for 20 minutes at 20 Volts. After that, the slides were neutralized using neutralization buffer and then stained with ethidium bromide dye. Finally, these slides were seen under a fluorescence microscope and DNA damage was quantified (Singh et al., 1988). To evaluate the presence of micronuclei in erythrocyte cells, fresh blood without anticoagulant was used to prepare a thin blood film on clean microscopic glass slides separately from each bird and micronucleus assay technique was used to analyze the frequency of micronuclei in erythrocytes of treated and normal birds according to the earlier described procedures (Rani et al., 2023; Riaz et al., 2025).

### Histopathology

Five birds were selected on a random basis from each group and were killed by cutting their jugular vein. Then necropsy was performed and different visceral organs (liver, kidneys and spleen) were extracted, weighed, and preserved in 15% paraformaldehyde solution. Subsequently, these preserved visceral organs were subjected to a histological examination. For histopathological observations, 4-5 µm thick slices were obtained by using a rotary microtome, followed by a dehydration process in alcohol, cleaning in xylene and staining with Hematoxylin and Eosin (Hussain et al., 2011).

### Statistical analysis

The collected data underwent statistical analysis using an analysis of variance test. The means of the groups were then compared through Tukey's test. The statistical software package used for this analysis was M-Stat. The probability level was set at or below (P ≤ 0.05).

## Results

### Clinical signs and behavioral alteration

During the study, various clinical and behavioral ailments were observed in Japanese quails of groups (B-D). No mortality occurred during the study. All the birds remained active and did not exhibit any clinical and behavioral alterations in acetochlor treated groups at days 15 of experiment. However, in group B very mild while in groups C and D, moderate to severe clinical and behavioral signs like depression, watery feces, dullness. anorexia, decline in the frequency of mounting with pen mates, reduced frequency of crowing, and decreased foam production was observed in Japanese quails at days 30 and 45 of experiment. The severity of various clinical and behavioral signs is indicated in Table 1.

**Table 1:** Overall severity of clinical and behavioral ailments.

Clinical ailments/days	Experimental groups			
	A	B	C	D
Depression	–	+	++	+++
Watery feces	–	+	+++	++++
Dullness	–	+	+++	++++
Anorexia	–	+	++	+++
Decline frequency of mounting with pen mates	–	+	+++	++++
Reduced frequency of crowing	–	+	++	++++
Decreased foam production	–	+	+++	++++

Notes: No clinical signs (–), very mild (+), moderate (++), severe (+++) and very severe (++++) were observed in quails of groups (B-C) treated with acetochlor.

### Status of enzymatic antioxidants and Oxidative stress parameters in liver

**Fig. 1:**
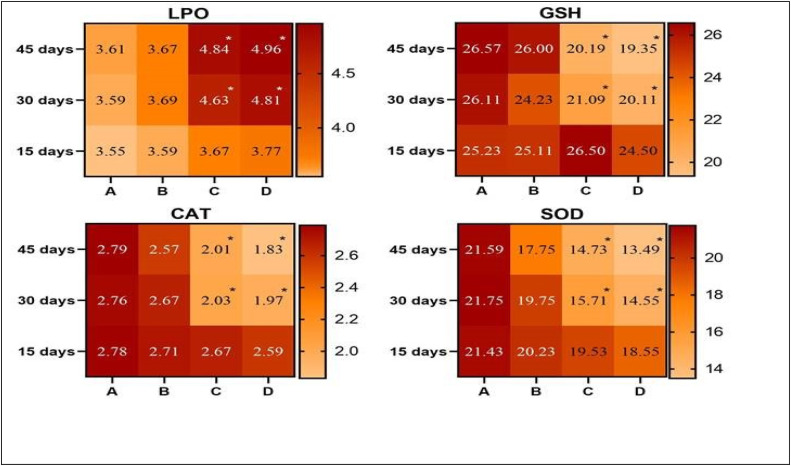
Photographs exhibiting comparison (using post hoc Tukey’s test at p < 0.05) of oxidative stress profile and status of enzymatic antioxidants in liver of male Japanese quails (n = 5) administered various concentrations of acetochlor at days 15, 30 and 45 of experiment. Values bearing (*) show significant difference compared to control group.

On days 30 and 45 of the experiment, there was a significant (P ≤ 0.05) increase in the contents of LPO in the hepatocytes of male Japanese quails placed in groups C and D. In contrast, the quantity of CAT, SOD, and GSH decreased significantly (P ≤ 0.05) in the hepatocytes of male Japanese quails reared in groups C and D as indicated in Fig. 1.

### Genotoxicity of the kidney, liver, and intestine

**Fig. 2:**
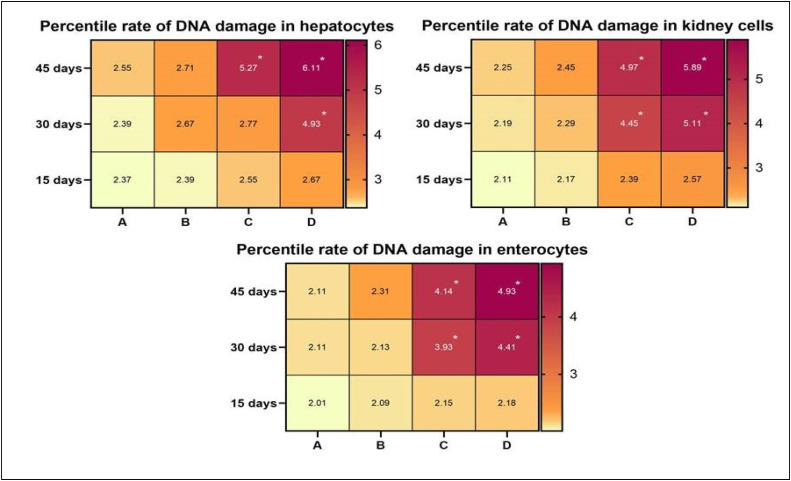
Photograph exhibiting comparison (using post hoc Tukey’s test at p < 0.05) of genotoxicity effects/percentile rate of DNA damage in isolated cells of hepatocytes, kidneys and intestine (enterocytes) of male Japanese quails (n = 5) administered various concentrations of acetochlor at days 15, 30 and 45 of experiment. Values bearing (*) show significant difference compared to control group.

**Fig. 3:**
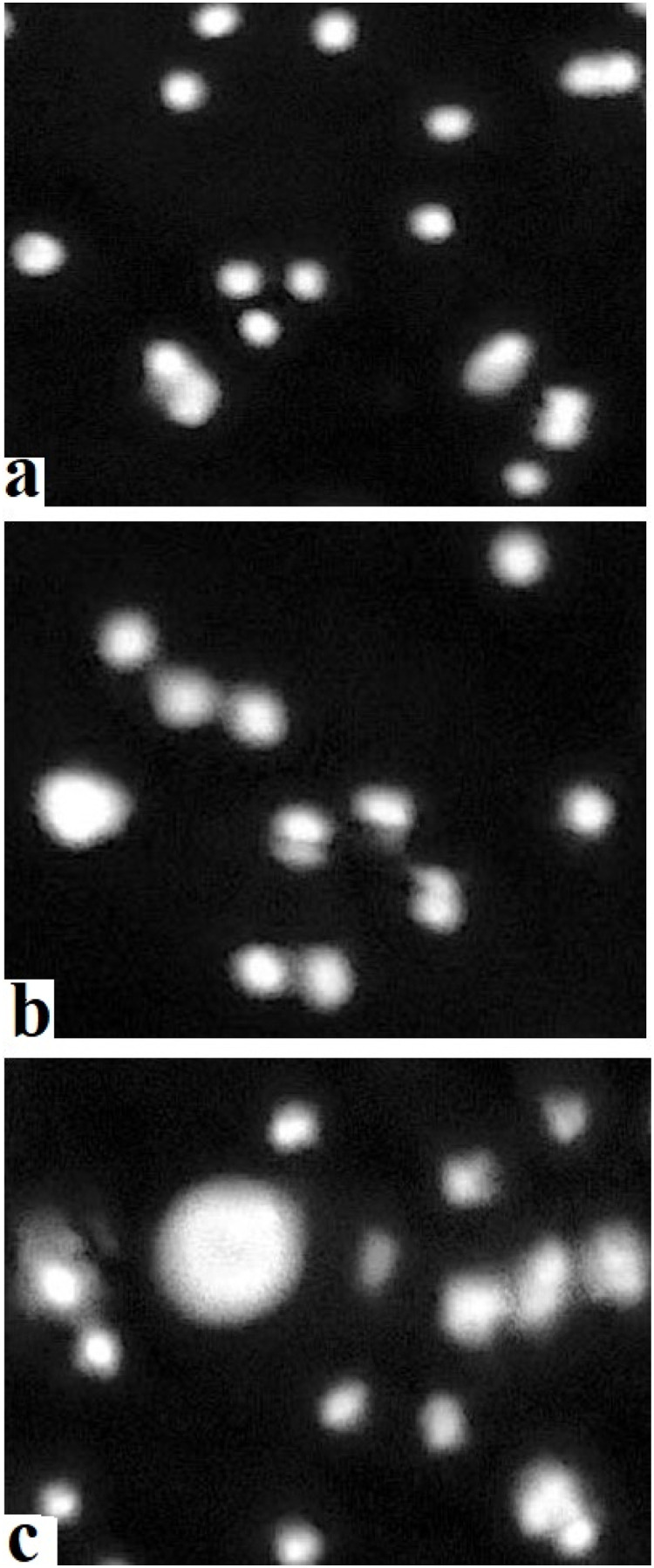
Comet assay/single cell gel electrophoresis assay indication DNA damage in isolated cells of different visceral organs including liver (a), kidneys (b) and intestinal cells (c) of quail exposed to higher doses (30 and 40 mg/kg) at day 45 of trial. Ethidium bromide stain. X400.

**Fig. 4:**
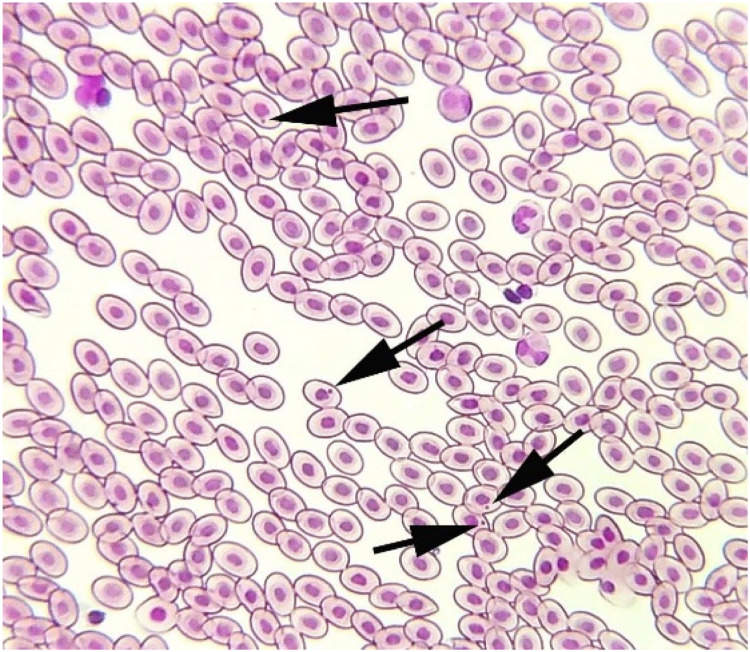
Thin blood smear showing different pathological ailments such as formation of micronuclei (arrows) in erythrocyte of quail exposed to higher doses (30 and 40 mg/kg) at day 45 of trial. Field stain A&B. X400.

The results on comet assay indicated a notable increase in the rate of DNA damage in isolated cells of different visceral organs (liver, kidneys, and intestine) of male Japanese quails on days 30 and 45 of the study (Fig. 2). The genotoxic potential of acetochlor remarkably increased in isolated cells of kidneys and enterocytes of male Japanese quails of groups C and D on days 30 and 45 of the experiment. Additionally, the DNA damage in hepatocytes of Japanese quails of group D increased significantly on day 30 of the study. Moreover, the frequency of mutagenic potential in isolated hepatocyte cells of quails of groups C and D was considerably higher when compared to quails of control group on days 30 and 45 of the study (Fig. 3). The results on formation of micronuclei in erythrocytes (Fig. 4) showed significantly higher percentile rate of presence of micronuclei in erythrocytes of quails reared in group D at day 30 while in groups (C-D) at day 45 of trial compared to untreated quails.

### Histopathology of liver, kidneys and spleen

**Fig. 5:**
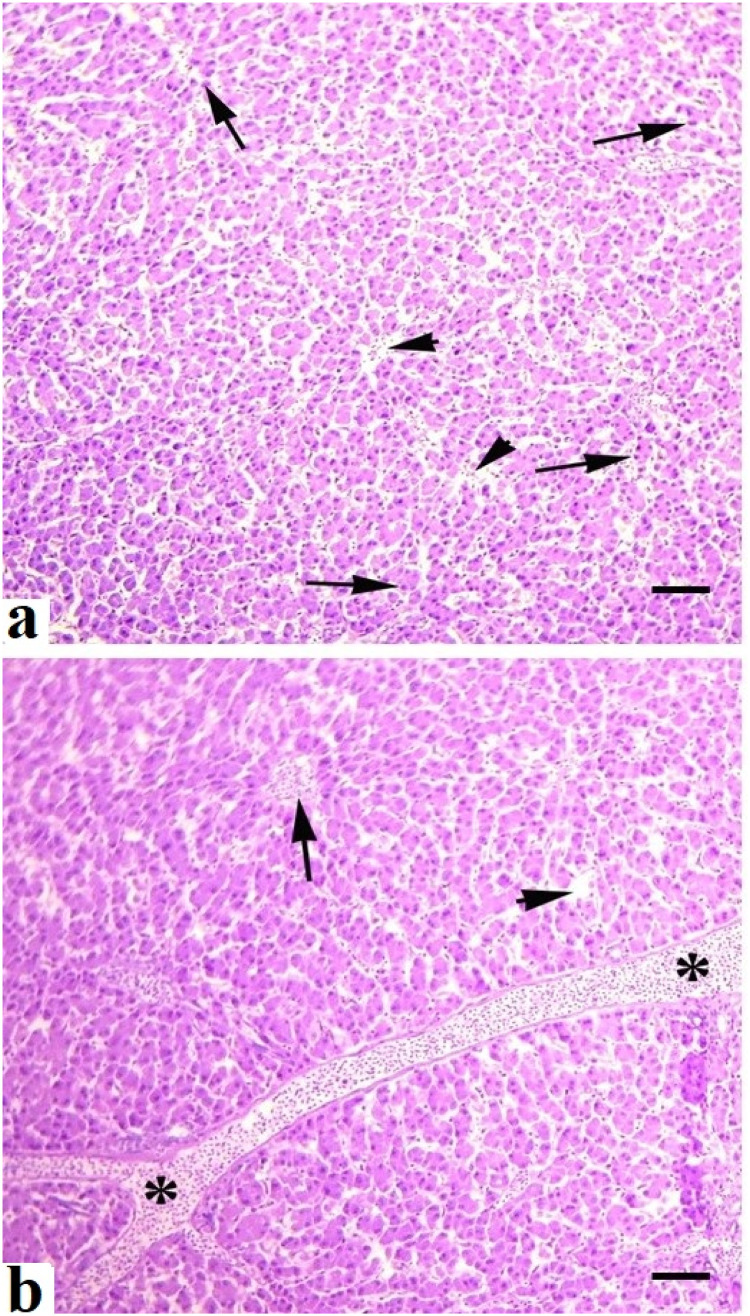
Histopathology of liver showing different pathological lesions like a) necrosis and degeneration of hepatocytes (arrows), disorganization and disruption of hepatic cards (arrow heads) and b), hemorrhages (*) and edema (arrow) and disruption of hepatic cards (arrow head) in quail exposed to higher doses (a; 30 mg/kg) and (b;40 mg/kg) at day 45 of trial. H&E stain. X400.

**Fig. 6:**
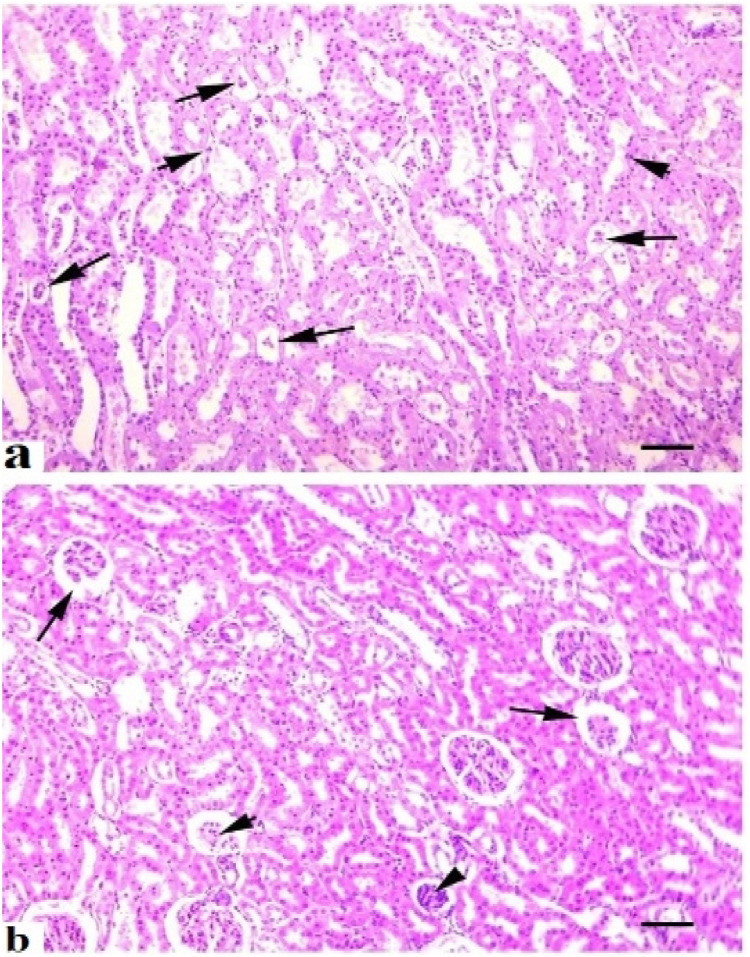
Histopathology of kidneys showing different pathological lesions a) necrosis and degeneration of renal tubular cells (arrow head), disorganization and degeneration of renal tubules (arrows) and b) widening of urinary space (arrows), necrosis of renal tubules (arrow heads) in quail exposed to higher doses (a; 30 mg/kg) and (b;40 mg/kg) at day 45 of trial. H&E stain. X400.

**Fig. 7:**
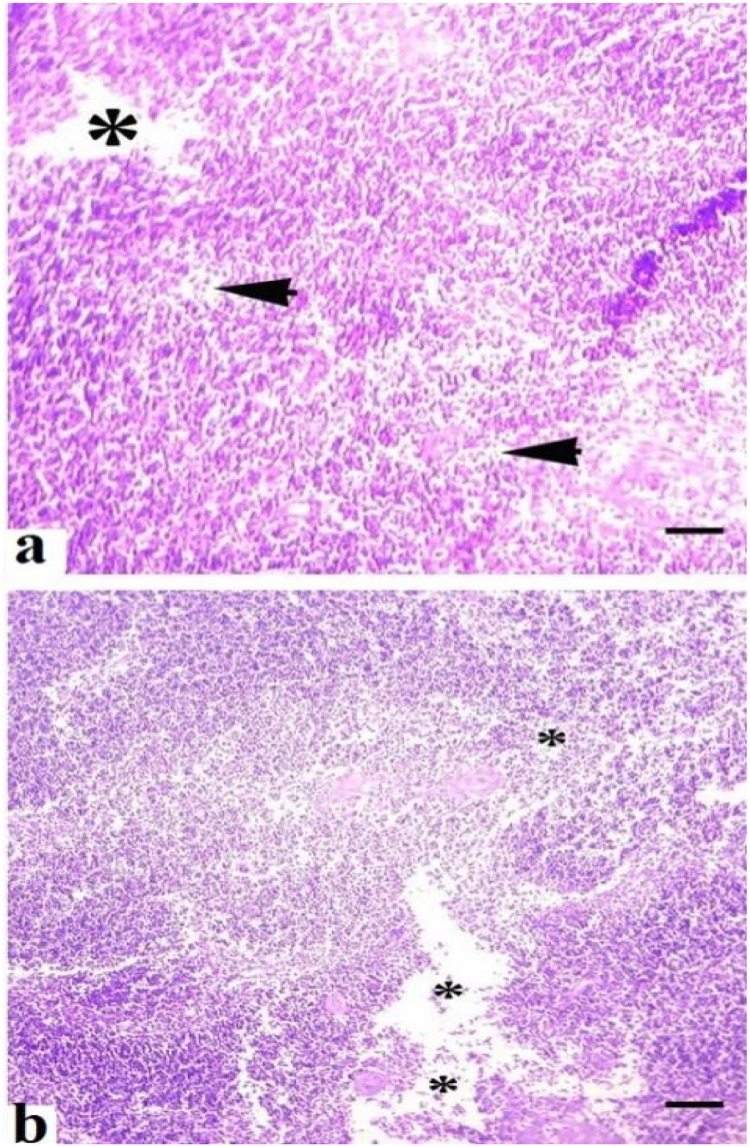
Histopathology of spleen showing different pathological lesions a) necrosis, degeneration and depletion of splenic cells (*) disorganization of splenic cells (arrow heads) and b) disorganization of white and red pulp (*) in quail exposed to higher doses (a = 30 and b = 40 mg/kg) at day 45 of trial. H&E stain. X400.

Grossly, the consistency, color, structure, shape, and texture of the liver, kidneys and spleen of quails of control group (A) were normal. No gross changes were observed in the liver, spleen and kidneys of birds kept in group B treated with the lower dose of acetochlor. Grossly, different prominent changes, including pale color, congestion, consistency, and texture of liver and kidneys of quails reared in groups (C and D) and exposed to higher doses of acetochlor, were observed. Mild to moderate congestion and increased size of spleen of quails treated with higher doses of acetochlor were observed at days 30 and 45 of the experiment. At light microscopic analysis, different moderate to severe histopathological alterations in various sections of liver like congestion, vacuolar degeneration, atrophy of cytoplasm of hepatocytes, necrosis, and widened sinusoidal spaces (Fig. 5) were examined the treated birds of groups C and D. Light microscopic analysis of various sections of kidneys of quails of groups C and D treated with higher doses of acetochlor indicated moderate to severe changes like edema, glomerular disintegration, ceroid formation, necrosis of renal tubules, widening of urinary space, and congestion (Fig. 6). At microscopic level, different sections of spleen of quails treated with higher doses of acetochlor exhibited disorganization of splenic cells, necrosis, degeneration and depletion of splenic cells and disorganization of white and red pulp (Fig. 7).

## Discussion

Acetochlor is a member of the chloroacetamide herbicide group, which is commonly used to control different weeds such as annual gramineae and small-seed broad-leaf plants in various food crops such as soybean, corn and sunflower (Hasan et al., 2021). It has been reported that when acetochlor is released in the atmosphere, it undergoes an oxidation reaction producing isocyanic acid, which is a potentially harmful compound (Wu et al., 2023). In previous studies, it is reported that the photodegradation products of acetochlor are known to have higher toxicity as compared to many other pesticides with a high potential for ecotoxicity (Zhang et al., 2023). No mortality was recorded in quails during the present experiment. Different clinical signs and behavioral changes, including decreased frequency of crowing, decline in frequency of mounting with pen mates, less foam production, dullness, depression and anorexia were observed in Japanese quails placed in experimental groups (B-D). Previously, no data were found regarding the toxic effects of acetochlor on these clinical signs and behavior alterations in Japanese quails. However, various clinical ailments, including nervous signs, behavioral and digestive alterations in quails treated with sub-lethal doses of lufenuron have also been observed in quails (Saeed et al., 2025).

In a previous study, it has been reported that the exposure to higher doses of insecticides resulted in a significant (P ≤ 0.05) increase in the level of LPO in the liver of quails while decreasing significantly (P ≤ 0.05) the contents of different antioxidant enzymes like CAT, SOD, and GSH (Saeed et al., 2025) in Japanese quails. The higher contents of biomarkers of oxidative stress and lower quantity of antioxidant enzymes in quails in this study might be linked to the induction of oxidative stress in terms of rapid and over-generation of free radicals by acetochlor. The increased contents of oxidative stress in our study have also been reported previously due to the negative impacts of toxic compounds in various exposed animals (Afzal et al., 2022; Fatima et al., 2025; Naseer et al., 2025). Lipid peroxidation (LPO) is a well-known free radical-mediated process responsible for oxidative degradation of polyunsaturated fatty acids, leading to rapid and over production of reactive oxygen species, ultimately causing injury and damage to cellular structures of various cells and tissues (Li et al., 2025). In previously published literature, different reports have indicated that insecticides, herbicides and pesticides induce oxidative stress via impairments and disruption of normal physiological functions, resulting in cellular and nuclear changes in avian species (Hussain et al., 2014; Afzal et al., 2024).

It is speculated that different antioxidant enzymes (CAT, SOD and GSH) are frequently measured to determine the adverse impacts of variety of toxic chemicals, while MDA and 8-iso-prostaglandin F2α (8-iso-PGF2α) are known as important biomarkers of the lipid peroxidation process (Li et al., 2018; Saeed et al., 2025). Enzymatic and non-enzymatic antioxidants play an important role in the maintenance of oxidative stress balance and redox reactions and provide defense against ROS formation (De Oliveira et al., 2021). Pesticides and herbicides have been known to induce ROS and cause oxidative stress in fish, cockerels and quails (Hussain et al., 2021; Veedu et al., 2022; Alam et al., 2025; Saeed et al., 2025). Antioxidant enzymes, including CAT, SOD and GSH are crucial in inhibiting oxidative stress and are often used to monitor the risk of pesticides/herbicides. Glutathione is also an appropriate biomarker for assessing the effect of pesticides/herbicides on various organisms (Raza et al., 2022; Saeed et al., 2025). In the present experimental study, significantly lower contents of different antioxidant enzymes such as GSH, SOD, and CAT in visceral organs (liver and kidneys) of quails given higher doses of acetochlor were measured. The significantly lower concentrations of antioxidant enzymes can be due to the inflammatory process leading to the generation of free radicals and depletion of antioxidant enzymes in quails. Various previous studies have indicated that anti-oxidant enzymes are useful and reliable biomarkers to determine the inflammation responses initiated via overproduction of reactive oxygen species (Saeed et al., 2025).

The results on comet assay/single cell gel electrophoresis assay showed a significant (P ≤ 0.05) increase in frequency of DNA damage in visceral organs (including the kidneys, liver and intestines) of the male Japanese quails exposed to higher concentrations of acetochlor. This dose-dependent DNA damage could be due to oxidative stress caused by acetochlor in Japanese quails. There is limited research on the genotoxic potential effects of acetochlor in the birds. However, some studies have suggested that acetochlor exposure may lead to DNA damage in birds. Previously, a study conducted on bighead carp exposed to acetochlor showed a significant increase in the rate of DNA damage in various tissues (Mahmood et al., 2022). The induction of genotoxic effects in this study in terms of significantly (P ≤ 0.05) increased DNA damage in multiple visceral organs and increased frequency of formation of micronuclei in erythrocytes of quail, might be due to abnormal interactions between plasma cholinesterase (BChE) activity and paraoxonase-1 (PON1) on catalase, glucose-6-phosphate dehydrogenase and glutathione peroxidase activities in erythrocytes. Significantly increased frequency of genetic damage in isolated cells of liver and kidneys and in erythrocytes of quails treated with acetochlor could be related to activation of caspase enzymes leading to over-release of apoptotic factors, nitration of nuclear proteins and methylation of genomic material (Afzal et al., 2024; Saeed et al., 2025). Moreover, the genotoxic effects caused by acetochlor could also be due to disorders in the cytochrome P450s gene and glutathione S-transferases in treated birds, resulting in pathological changes in different visceral organs (Mahmood et al., 2022). The increased frequency of DNA damage/genotoxicity can also be related to genetic variants causing lower metabolic activity in treated quail (Qamar et al., 2023). Histopathological analysis revealed congestion, atrophy and degeneration of cytoplasm of hepatocytes, vacuolar degeneration, necrosis of hepatocytes, and widened sinusoidal spaces in the hepatocytes in quails treated with higher doses of acetochlor. Previously, similar microscopic lesions like necrosis of hepatocytes, fatty changes and atrophy in the liver of *Clarias gariepinus* and bighead carp have also been observed (Hassan et al., 2023). Moreover, acetochlor exposure induced histopathological ailments in the kidneys of quails like edema, ceroid development, glomerular disintegration, and congestion. Previously, similar histopathological ailments in kidneys of *Ctenopharyngodon idellus* (Zhao et al., 2022), while necrosis, vacuolation, accumulation of melano-macrophages, congestion, and other tissue changes have been observed in *Heteropneustes fossilis* and tilapia (Vinodhini and Narayanan, 2009), big head carp and *Ctenopharyngodon Idella* (Li et al., 2022) exposed to acetochlor and other toxicants. Previously, sinusoidal dilation, tubular degeneration, necrosis of tubular epithelial cells, increased urinary space and hemolysis in the kidneys of different avian species pesticide toxicity have also been observed (Hussain et al., 2011; Hussain et al., 2021; Gul et al., 2025). Various microscopic changes in the spleen of quails in the present study, such as disorganization of splenic cells, necrosis, degeneration and depletion of splenic cells and disorganization of white and red pulp could be due to oxidative stress as indicated by increased contents of oxidative stress biomarkers in this study. Histo-pathological alterations in liver, kidneys, and spleen of acetochlor treated quails, including necrotic lesions, might be due to the induction of oxidative stress in terms of increased production and release of reactive oxygen species that damage the cellular and biological membranes.

## Conclusion

In conclusion, the study demonstrates that exposure to higher doses of the herbicide Acetochlor leads to clinical symptoms, behavioral changes, impaired antioxidant defense, increased oxidative stress, DNA damage, and histopathological alterations in the vital organs of male Japanese quails. Specifically, quails administered higher Acetochlor concentrations showed symptoms of dullness, depression, and anorexia, along with declines in mating behaviors. Additionally, the livers exhibit weakened enzymatic antioxidant activity, elevated lipid peroxidation, and increased genotoxicity. Furthermore, histopathological examinations reveal structural abnormalities in the liver and kidneys of treated quails, indicative of cellular damage. Collectively, these deleterious effects intensify with increasing Acetochlor dosage, affirming the herbicide's dose-dependent toxicity in male Japanese quails. Further studies can assess its impact on female quails and other avian species.

## Data availability

All relevant data are within the manuscript file.

## CRediT authorship contribution statement

**Dong Shiqi:** Conceptualization, Project administration, Software, Methodology, Data curation, Writing – original draft. **Mohammed Al-Rasheed:** Writing – original draft, Software, Project administration, Resources, Data curation, Conceptualization. **Sahar Faiz Lodhi:** Conceptualization, Writing – original draft, Validation, Methodology, Data curation. **Nuzhat Sial:** Investigation, Data curation, Methodology, Conceptualization. **Riaz Hussain:** Writing – original draft, Supervision, Software, Data curation, Investigation, Conceptualization. **Maha Abdullah Momenah:** Conceptualization, Writing – review & editing, Software, Data curation, Project administration. **Mahmoud H.A. Mohamed:** Writing – review & editing, Data curation, Conceptualization. **Ahmed M.A. Meligy:** Conceptualization, Writing – review & editing, Resources, Formal analysis. **Mohamed S. Ahmed:** Writing – review & editing, Data curation, Conceptualization. **Ahmed Alfifi:** Writing – review & editing, Software, Conceptualization, Project administration. **Nahid Abdelraheem Ali:** Writing – review & editing, Conceptualization, Investigation. **Zhang Jiabin:** Conceptualization, Funding acquisition, Project administration, Resources, Writing – original draft.

## Disclosures

The authors declare that they have no known competing financial interests or personal relationships that could have appeared to influence the work reported in this paper.
